# Childhood psychopathology in children of women with eating disorders: understanding risk mechanisms

**DOI:** 10.1111/jcpp.12112

**Published:** 2013-06-27

**Authors:** Nadia Micali, Daniel Stahl, Janet Treasure, Emily Simonoff

**Affiliations:** 1 Behavioural and Brain Sciences Unit, Institute of Child Health, UCLLondon, UK; 2 Department of Biostatistics, King’s College London, Institute of PsychiatryLondon, UK; 3 King’s College London, Eating Disorders Research Unit, Institute of Psychiatry, De Crespingy ParkLondon, UK; 4 King’s College London, Child and Adolescent Psychiatry Department, Institute of Psychiatry, De Crespingy ParkLondon, UK

**Keywords:** ALSPAC, child psychopathology, eating disorders, parental mental health, risk mechanisms

## Abstract

**Background:**

Very few studies have investigated psychopathology in children of mothers with eating disorders (ED). We aimed to determine the effect of maternal ED on childhood psychopathology in a large population-based cohort and investigate relevant risk pathways using structural equation modeling (SEM).

**Methods:**

Data on emotional and behavioral problems at 3½ years were obtained prospectively on 8,622 children from the Avon Longitudinal Study of Parents and Children (ALSPAC). Children of exposed women who self-reported lifetime anorexia nervosa (AN, *N* = 193) or bulimia nervosa (BN, *N* = 158) in pregnancy were compared with children of unexposed women (*N* = 8,271) using linear and logistic regression models. SEM was used to determine best-fitting risk models by child gender.

**Results:**

There was evidence that girls of AN women were more likely to have emotional, conduct, and hyperactivity disorders [Odds Ratio (OR): 1.7 (95% Confidence Intervals 1.0–3.0); OR: 2.2 (1.2–4.0); OR: 1.8 (1.1–3.1), respectively] and boys of AN women to have emotional disorders compared with unexposed [OR: 2.0(1.2–3.4)]. Girls of women with BN were more likely to show hyperactivity [OR: 1.7 (1.0–3.1)]; and boys to show emotional and conduct disorders compared with unexposed [OR: 2.2 (1.2–3.9); OR: 2.4 (1.4–4.2), respectively]. SEM models showed that pregnancy anxiety and depression mediated the effect of maternal ED on child psychopathology.

**Conclusions:**

Maternal ED are associated with different childhood psychopathology outcomes in boys and girls. Pregnancy anxiety and depression and active ED symptoms are important mediators of risk and are preventable; the direct effect of maternal lifetime ED was small.

## Introduction

Although the impact of parental psychiatric disorder on child development is well documented, parental eating disorders (ED) have been less researched than other disorders. Research on the effects of maternal ED has mainly focused on the homotypic (i.e., the development of ED in the offspring) rather than heterotypic effects (i.e., the development of other psychopathology). There is evidence that maternal disordered eating is associated with adolescent disordered eating (Field et al., 2008; Pike & Rodin, 1991); and maternal ED with eating problems in childhood (Micali, Simonoff, & Treasure, 2009; Stein, Woolley, Cooper, & Fairburn, 1994; Stein et al., 2006). Few studies, however, have investigated the effects of maternal ED on childhood psychopathology. Focusing on children at risk for ED (those born to women with ED) can help not only understand the effects of a maternal disorder but can also help clarify potential etiological factors associated with the risk for ED, including shared liability with other psychiatric disorders.

Two very small early studies investigated the presence of psychological disturbance in children of mothers with ED: one studied 19 children, of which 5 had behavioral problems (Franzen & Gerlinghoff, 1997); the other studied 27 children whose mothers suffered with an ED finding high levels of psychological disturbance (17–29%) (Timimi, 1996).

A more recent follow-up comparison of 10-year-old children born to women with ED (*N* = 33) and controls (*N* = 23) found weak evidence of a difference between index children and controls in emotional symptoms reported by teachers (Stein et al., 2006).

These findings (Franzen & Gerlinghoff, 1997; Stein et al., 2006; Timimi, 1996) have been confirmed in a large general population study showing higher anxiety scores in 3-year-old children of mothers with ED compared with children of non-ED women (Reba-Harrelson et al., 2010).

If the above findings bear true, little is known on the mechanisms explaining this higher risk in the offspring of ED women. Both genetic and environmental risk factors might be responsible; within environmental factors, disentangling in utero effects versus postnatal effects is important for adequate prevention.

The in utero effects of parental psychopathology have received much interest in the last decade (Rice, Jones, & Thapar, 2007). Increasing evidence suggests a role for fetal programming in contributing to the risk for neurodevelopmental disorders and psychopathology (Bale et al., 2010). The effect of prenatal stress and anxiety on childhood behavioral and emotional symptoms has been established across human studies and animal research (for a review, see Glover, 2011).

Perinatal events, especially obstetric complications, have been associated with several psychopathological outcomes, both in adults and children: schizophrenia, autism, and attention deficit hyperactivity disorder (ADHD) as well as ED (Nosarti et al., 2012).

Gender differences in childhood psychopathology have been shown and understanding these in the context of risk mechanisms has been highlighted as important in aiding identification of causal processes (Rutter, Caspi, & Moffitt, 2003). Moreover, there is evidence of a differential effect (by gender) of parental psychopathology on offspring psychopathology in early childhood (Ramchandani et al., 2008).

No previous study has explored the whole range of psychopathology (behavioral and emotional symptoms) in children of women with ED; moreover, the in utero risk mechanisms that might be partially responsible for an increased risk in psychopathology have not been investigated.

We used data from a large general population study, the Avon Longitudinal Study of Parents and Children (ALSPAC) to address some of the unanswered questions on heterotypic risk for psychopathology in children of women with ED and to model risk, focusing on preventable maternal in utero and child mediators.

We aimed to: (a) investigate psychopathology, i.e., levels of emotional, conduct, hyperactivity symptoms, and total psychopathology, in children of women with lifetime ED at age 3½ years compared with children of unexposed women; (b) understand potential pregnancy maternal factors that might mediate the risk for childhood emotional, conduct, or hyperactivity problems in female and male children of women with ED.

Given the evidence that pregnancy maternal anxiety and depression (Bergman, Sarkar, Glover, & O’Connor, 2008; O’Connor, Heron, Golding, & Glover, 2003) predict child psychopathology, and have a stronger effect than postnatal psychopathology (O’Connor et al., 2003) and the high levels of prenatal emotional disorders in women with ED (Micali, Simonoff & Treasure, 2011), we focused on the effect of maternal pregnancy anxiety and depression. We were also interested in disentagling the effect of lifetime maternal ED and active ED symptoms in pregnancy (to focus on in utero effects). Given our previous findings on the effect of maternal ED on birthweight (Micali, Simonoff, & Treasure, 2007), and known child risk factors for childhood psychopathology, such as temperament, developmental status, life events, and parenting (Caspi & Shiner, 2009; Rutter & Sroufe, 2000), we also investigated these as mediators/independent predictors.

## Methods

### Sample

The Avon Longitudinal Study of Parents and Children is a longitudinal, population-based, prospective study of women and their children (Golding, Pembrey, & Jones, 2001). All pregnant women living in the geographical area of Avon, UK, who were expected to deliver their baby between 1 April 1991 and 31 December 1992, were invited to take part in the study. All women gave informed and written consent. The sample has been shown to be representative of the local population; 14,663 women were enrolled at the 9th week of pregnancy; data were obtained on 14,472 women via postal questionnaires. Only singleton births were included in this study (14,273). Women were excluded from this study if they had not answered the questionnaire sent at approximately 12 weeks (2,019) enquiring about lifetime psychopathology.

Of the available 12,254 subjects, 12,130 children were alive at 1 year. Of these 2,686 did not complete the questionnaire on the outcome measure; therefore, 9,444 mothers and children (78%) were available for this study. We excluded from the analyses women who reported lifetime psychiatric disorders only (other than ED) at 12 weeks (822).

The final sample available was 8,622 women and their children.

### Measures

#### Sociodemographic data

Maternal age, parity, maternal education, maternal weight and height preconception, and ethnicity were obtained at recruitment or during pregnancy.

#### Main predictor

Maternal lifetime ED was determined using two questions: at 12 weeks, all women were asked whether they had a history of Anorexia Nervosa (AN) and/or Bulimia Nervosa (BN): 193 women reported a history of AN; 158 of BN; and 81 both. Women with lifetime AN and those with both AN and BN were grouped together, after preliminary analyses showing similar baseline characteristics (for details, see Micali et al., 2007) and similar effects on outcomes under study. There is good evidence that self-report of ED is sensitive and specific (Keski-Rahkonen et al., 2007; Micali et al., 2012).

#### Outcome

Childhood psychopathology: mothers reported on their child’s behavior using the Strengths and Difficulties questionnaire (SDQ) at 3½ years (Goodman & Scott, 1999). The SDQ is a widely used instrument to assess childhood psychopathology translated into more than 40 languages. It has been validated against several other well-established instruments assessing childhood psychopathology. The SDQ includes 25 items and 5 subscales, of which 4 were used in ALSPAC: emotional problems, conduct problems, inattention/hyperactivity, and prosocial behavior; it also generates a total difficulties score (Goodman, 1999). All SDQ subscales have good internal consistency in this sample (Cronbach’s alphas >0.62).

For this study, we used the emotional problems, conduct problems, inattention/hyperactivity subscales, and the total difficulties score. Emotional, conduct, and inattention/hyperactivity scores were not normally distributed and could not be normalized. Therefore, all scores were dichotomized: high scorers were identified using a cut-off of the top 10% scores on each subscale (using a score of ≥5 for the inattention/hyperactivity and emotional subscales, of ≥7 on the conduct subscale, and of ≥20 on the total difficulties scale), the bottom 90% served as the nonsymptomatic group. These cut-offs were chosen following a sensitivity analysis carried out using SDQ scores at later time points (81 and 97 months), which showed stability of the chosen cut-offs across the three time points; published SDQ cut-offs for 5- to 10-year-old British children and 3- to 4-year-old Spanish children (Ezpeleta, Granero, la Osa, Penelo, & Domènech, 2013; Goodman, 1999). The top 10% cut-off has been used in previous research using the SDQ at older ages (Plomin, Price, Eley, Dale, & Stevenson, 2002) and in this sample at this age (Ramchandani et al., 2008) and shown to index clinical pathology.

### Other risk factors and mediators:

#### Maternal

Anxiety: measured at 32 weeks in pregnancy, and 33 months postpartum using the Crown Crisp Experiential Inventory (CCEI) (Crown & Crisp, 1978). In this sample, internal consistency of this measure was >0.8.

Depression: measured at 32 weeks in pregnancy, and 33 months postpartum using the Edinburgh Postnatal Depression Scale (EPDS) (Cox, Holden, & Sagovsky, 1987) (for details on both measures, see Micali, Simonoff, & Treasure, 2011). Internal consistency for this measure in ALSPAC was >0.8.

ED symptoms in pregnancy: investigated at 18 weeks in pregnancy using the shape and weight concern subscales of the Eating Disorders Examination Questionnaire (EDE-Q) (Fairburn & Beglin, 1994) and questions about compensatory behaviors (i.e., self-induced vomiting and laxative use for weight loss) (Micali et al., 2007). A binary variable was created to account for ED symptomatology in pregnancy: women with a score of >2 on the shape and weight concern subscales (each consisting of five items) of the EDE-Q (a recognized and previously used cut-off) (Stein, Stein, Walters, & Fairburn, 1995) and/or the presence of compensatory behaviors were defined as having *pregnancy ED symptoms* (coded as 1).

#### Child

Temperament: measured at child age 6 months by the Carey infant temperament scale. This scale generates several subscales, each measuring a temperamental trait; it has been shown to have high test–retest reliability and internal consistency (Carey & McDevitt, 1978). Mothers were given statements describing their child’s behavior and asked to rate how often their child behaved in that way on a likert scale from 1 (almost never) to 6 (almost always). We used the mood, intensity, adaptability, approach, and rhythmicity subscales and summed these to obtain a continuous variable indicating ‘difficult’ temperamental traits (Micali, Simonoff, Stahl, & Treasure, 2011); according to the Thomas & Chess definition of ‘difficult temperament’ (Chess & Thomas, 1977).

Developmental status: The mother-completed version of the Denver Developmental scale on their 2½-year-old children (Frankenburg & Dodds, 1967) was used (28 items, covering 3 areas: social, fine motor, and gross motor development); a total score was obtained and used as a continuous variable. This questionnaire was validated in an ALSPAC subsample against a gold standard (the Griffiths scale administered under controlled conditions by trained psychologists) with a correlation coefficient of 0.54 (*p* < .0001).

Life events: assessed by a questionnaire completed by mothers at child age 2½, recording whether the child had experienced any of 16 upsetting events in the year before. The items included were taken from previous studies (Barnett, Hanna, & Parker, 1983; Brown & Harris, 1978). The total number of events in the previous 12 months was used as a categorical score due to nonnormality (none, 1–3, 4, or more).

#### Obstetric

Birthweight was obtained from obstetric records.

#### Parenting

Measured when the child was 2 by questionnaire to mothers. Mothers were asked to indicate the frequency of doing a set of activities with their children. A score was obtained by summing responses to these questions. Higher scores indicated higher frequency of engaging in positive activities with the child. This score has been shown to predict child development and to be associated with socio-economic factors in ALSPAC (Lawson & Mace, 2009).

All measures were extensively piloted by the ALSPAC study team and have been used in previous studies; for details, see www.ALSPAC.bris.ac.uk.

### Ethics

The study was approved by the Institute of Psychiatry Ethics committee (Ref. 110/02), the ALSPAC Law and Ethics committee and the Local Research Ethics Committees.

### Data Analyses

Data analysis was carried out in four stages:

1. Initially the outcomes of interest: binary SDQ subscales and full-scale scores were compared across exposure groups separately by gender in unadjusted and adjusted analyses. Outcomes found to be associated with each exposure were chosen for further investigation of causal pathways.
2. The role of selected maternal and child factors hypothesized as possible mediators was then investigated in univariable logistic regression analyses.
3. If a variable hypothesized to be a mediator based on previous research met statistical criteria for this [i.e., they were associated with predictor and outcome; they were situated on the causal pathway between the predictor and the outcome (Kraemer, Stice, Kazdin, Offord, & Kupfer, 2001)], a structural equation model (SEM) was built to test how the model fit the available data. If a variable was shown to be only associated with the outcome, it was analyzed as an independent predictor in the SEM model.
4. A model per outcome and per gender was built by successive iterations. Variables were entered in the successive models one-by-one. Those (mediators or independent) predictors nearer exposure (in time) were entered first (pregnancy ED symptoms and depression/anxiety at 32 weeks gestation); obstetric ones (birthweight) were entered at a second stage; postpartum variables (maternal anxiety and depression at 33 months post-partum and parenting) at a third stage; child factors (life events, development, and temperament) at a fourth stage. Eight SEM models for each outcome (and for each gender) were tested. Models were assessed for good fit to the data and the model that best fits the data was then chosen as the final model.

### Structural equation modeling and model testing

The hypothesized models were tested using SEM. SEM is a method to describe, assess, and test hypothesized relationships between observed (manifest or unmeasured) variables and unobserved (or latent) variables. SEM is commonly graphically represented: observed variables are enclosed by rectangles and latent variables by circles. An assumed causal path between two variables is shown by a directed edge (single-headed arrow).

Path coefficients on the edges are partial standardized regression coefficients, which measure the effect of one variable on another; while controlling for all other variables prior in the model, all coefficients show a positive association, but those with a minus sign indicate a negative association. Parameters were estimated using Full Maximum Likelihood methods, which make use of all available data and provide unbiased and efficient parameter estimates if data are missing at random (Allison, 2001), whereas traditional methods using listwise deletion require more strict assumptions about missingness (missing completely at random) and have less power (Allison, 2001).

The Root Mean Square of Approximation (RMSEA, a parsimony-adjusted index) and the Comparative Fit Index (CFI) were used for model comparison and final model selection. For each set of analyses, the model with the lowest RMSEA was chosen as best fitting the data.

For each gender, we fitted separate parameters in the model and did not constrain any to be the same among groups.

### Attrition

We investigated patterns of missingness for missing data on the SDQ at 3½ years (*n* = 2,686) among all children who had data on the exposure under study using a binary indicator in logistic regression models; there was no evidence of selective attrition across exposure groups.

Attrition at age 3½ was associated with lower maternal age and maternal education and higher parity. All variables predicting attrition were included in multivariable analyses to fulfill the assumptions of missing at random.

Stata 11 (Stata Corp., TX) was used for data manipulation and regression analyses. We used the computer program Mplus (version 6) (Muthén & Muthén, 2009) to carry out SEM.

## Results

### Sociodemographic data

Table 1 contains sociodemographic information on the studied sample. Women with lifetime AN were slightly older and more commonly educated to a-level or degree qualification.

**Table 1:** Sociodemographic data (relative to mother and child): comparisons based on ANOVA, logistic regression (in bold)

	AN (*N* = 193)	BN (*N* = 158)	Unexposed (*N* = 8,271)
Maternal variables			
Maternal age at delivery (years), mean (*SD*)	29.5** (4.9)	28.4 (4.3)	28.7 (4.6)
**Parity (multiparous)**	**52.1%**	**51.6%**	**54.4%**
**(OR, 95% CI)**	**(0.9, 0.7–1.2)**	**(0.9, 0.6–1.2)**	**Ref.**
**Ethnicity (white)**	**97.3%**	**98.0%**	**98.2%**
**(OR, 95% CI)**	**(0.7, 0.3–1.7)**	**(0.9, 0.3–2.9)**	**Ref.**
**Education** (A-level and degree vs. up to GCSE)	54.0%***	**39.6%**	**39.3%**
**(OR, 95% CI)**	**(1.8, 1.3–2.4)**	**(1.0, 0.7–1.4)**	**Ref.**
EPDS in pregnancy mean (*SD*)	9.3*** (6.1)	8.8*** (5.5)	6.5 (4.7)
CCEI in pregnancy, mean (*SD*)	19.2*** (9.5)	19.7*** (9.1)	14.3 (7.7)
Child variables			
**Child gender (male),%**	**52.3%**	**49.4%**	**51.3%**
**(OR, 95% CI)**	**(1.0, 0.8–1.4)**	**(0.9, 0.7–1.3)**	**Ref.**
Birthweight (in grams), mean (*SD*)	3414 (517)	3508 (500)	3443 (526) Ref.
Gestational age, mean (*SD*)	39.6 (1.8)	39.7 (1.6)	39.5 (1.8) Ref.
Difficult temperament, mean (*SD*)	89.3 (20.4)	89.1 (19.8)	86.2 (20.0)
Developmental score, mean (*SD*)	64.5 (10.1)	65.2 (8.1)	64.9 (7.6)
**No of life events: none, *n* (%)**	**42 (21.5%)**	**26 (16.0%)**	**2,519 (29.7%)**
**1–3, *n* (%)**	**132 (67.7%)**	**121 (74.2%)**	**5,426 (64.0%)**
**OR (95% CI)**	1.4* **(1.0–2.1)**	2.2*** **(1.4–3.3)**	**Ref.**
**4–15, *n* (%)**	**21 (10.7%)**	**16 (9.8%)**	**526 (6.2%)**
**OR (95% CI)**	2.4*** **(1.4–4.1)**	2.9*** **(1.6–5.5)**	**Ref.**

OR, Odds ratio; 95% CI, 95% confidence interval; EPDS, Edinburgh Postnatal Depression Scale; CCEI, Crown Crisp Experiential Inventory.

*p < .05; **p ≤ .01, ***p < .001 for comparisons between groups and the General Population.

### Childhood Psychopathology

Child gender was predictive of having conduct problems [males vs. females: odds ratio (OR) = 1.5, 95% confidence intervals (CI): 1.3–1.7, *p* < .0001], hyperactivity/inattention (males vs. females: OR = 1.4, 95% CI: 1.2–1.6, *p* < .0001), and total difficulties (males vs. females: OR = 1.3, 95% CI: 1.1–1.5, *p* < .0001); but not of having emotional problems (males vs. females: OR = 0.9, 95% CI: 0.8–1.1, *p* = .13).

Children of women with AN (both boys and girls) and male children of women with BN were more likely to have emotional problems (indexed by scoring on the top 10%) and children of women with BN were more likely to have conduct problems (see Table 2).

**Table 2:** Psychopathology across groups: percentages, crude, and adjusted odds ratios (OR) and 95% confidence intervals (95% CI) from logistic regression

	%, Crude OR (95% CI)			Adjusted^a^ OR (95% CI)		
	AN (*N* = 193)	BN (*N* = 158)	Unexposed (*N* = 8271)	AN (*N* = 193)	BN (*N* = 158)	Unexposed (*N* = 8271)
All						
Emotional problems	19.2% 1.8** (1.2–2.5)	18.3%	11.8%	1.9*** (1.3–2.7)	1.7** (1.2–2.6)	Ref.
		1.7** (1.1–2.5)				
Conduct problems	13.5% 1.3 (0.9–2.0)	18.3%	10.5%	1.4 (0.9–2.2)	2.1*** (1.4–3.2)	Ref.
		1.9** (1.3–2.9)				
Hyperactivity problems	17.6% 1.3 (0.9–1.8)	19.6%	14.5%	1.2 (0.8–1.8)	1.4† (0.9–2.1)	Ref.
		1.4† (1.0–2.1)				
Total difficulties	14.5% 1.5† (1.0–2.2)	17.1%	10.3%	1.7** (1.1–2.5)	1.8** (1.2–2.8)	Ref.
		1.8** (1.2–2.7)				
Boys						
Emotional problems	19.8% 1.9** (1.2–3.2)	20.5%	11.3%	2.0** (1.2–3.4)	2.2** (1.2–3.9)	Ref.
		2.0** (1.2–3.5)				
Conduct problems	11.9% 0.9 (0.5–1.8)	24.4%	12.3%	0.9 (0.5–1.7)	2.4*** (1.4–4.2)	Ref.
		2.3** (1.3–3.9)				
Hyperactivity problems	14.8% 0.9 (0.5–1.5)	20.5%	16.7%	0.9 (0.5–1.5)	1.2 (0.6–2.1)	Ref.
		1.3 (0.7–2.2–2.1)				
Total difficulties	12.9% 1.1 (0.6–2.0)	21.8%	11.5%	1.3 (0.7–2.3)	2.1** (1.2–3.7)	Ref.
		2.1** (1.2–3.7)				
Girls						
Emotional problems	18.5% 1.6† (0.9–2.7)	16.2%	12.4%	1.7* (1.0–3.0)	1.4 (0.8–2.6)	Ref.
		1.4 (0.7–2.5)				
Conduct problems	15.2% 1.9* (1.1–3.5)	12.5%	8.4%	2.2** (1.2–4.0)	1.7† (0.9–3.3)	Ref.
		1.5 (0.8–3.0)				
Hyperactivity problems	20.6% 1.9* (1.1–3.1)	18.7%	12.2%	1.8* (1.1–3.1)	1.7* (1.0–3.1)	Ref.
		1.7† (0.9–2.9)				
Total difficulties	16.3% 1.9* (1.1–3.5)	12.5%	8.9%	2.2** (1.3–4.0)	1.5 (0.8–3.0)	Ref.
		1.5 (0.7–2.8)				

a adjusted for maternal age, education, and parity.

*p ≤ .05; **p ≤ .01; ***p ≤ .001; †*p* < .1.

Girls of women with AN were more likely to have emotional, conduct, and hyperactivity problems compared with unexposed children (see Table 2). In contrast, girls of women with BN were more likely to have hyperactivity/inattention problems compared with unexposed (adjusted OR = 1.7, 95% CI: 1.0–3.1, *p* < .05).

Boys of women with AN were twice as likely to have emotional problems (adjusted OR = 2.0; 95% CI: 1.2–3.4, *p* < .01), whereas boys of BN women were twice as likely to have emotional and conduct problems (adjusted OR = 2.2, 95% CI: 1.2–3.9, *p* < .01; OR = 2.4, 95% CI: 1.4–4.2, *p* < .001, respectively) compared with children of unexposed women.

Given the gender differences highlighted above in gender-stratified analyses, we also carried out interaction tests and found a marginally significant interaction for gender × Maternal AN for hyperactivity problems (*p* = .06) and a significant gender × Maternal AN interaction for conduct problems (*p* = .04) and emotional problems (*p* = .04).

### Mediators and causal pathways

Given the associations above, we focused on maternal and child mediators of interest for these.

Table 3 shows univariable associations between maternal and child factors and the outcomes (emotional, conduct, and hyperactivity problems). All risk factors under study were associated with the outcomes.

**Table 3:** Maternal and child predictors for psychopathology at 3½: univariate logistic regression, odds ratios (OR), and 95% confidence intervals (CI)

	Emotional problems	Conduct problems	Hyperactivity problems
	OR (95% CI)	OR (95% CI)	OR (95% CI)
Maternal factors			
ED symptoms in pregnancy	1.4** (1.2–1.6)	1.6** (1.4–1.9)	1.4** (1.2–1.6)
Anxiety at 32 weeks gestation	1.06** (1.05–1.07)	1.05** (1.04–1.06)	1.04** (1.03–1.04)
Depression at 32 weeks gestation	1.08** (1.06–1.09)	1.08** (1.06–1.09)	1.05** (1.04–1.06)
Anxiety at 2.7 years postpartum	1.07** (1.06–1.08)	1.07** (1.06–1.08)	1.04** (1.03–1.05)
Depression at 2.7 years postpartum	1.08** (1.07–1.1)	1.08** (1.07–1.1)	1.06** (1.05–1.07)
Parenting score at 2 years	0.96* (0.94–0.99)	0.92** (0.90–0.94)	0.95** (0.94–0.98)
Child factors			
Developmental level at 2.5 years	0.97** (0.95–0.97)	0.98** (0.97–0.98)	0.96** (0.95–0.97)
Number of life events in past year (at 2.5 years)	1.3** (1.2–1.5)	1.5** (1.3–1.7)	1.3** (1.1–1.4)
Difficult temperament at 6 months	1.01** (1.01–1.02)	1.01** (1.01–1.02)	1.01** (1.01–1.02)
Birthweight in Kg	0.8** (0.7–0.9)	1.0 (0.9–1.1)	0.8** (0.7–0.9)

*p ≤ .005; **p ≤ .0001.

All factors were therefore included in SEM models, and their role was investigated as mediators [if criteria for being a mediator were met (see above)], or independent predictors (if they were associated with the outcome only).

### Girls

We investigated causal mechanisms for girls of women with AN for emotional, conduct, and hyperactivity problems. Girls of women with BN were studied in relation to hyperactivity only.

#### Emotional problems in girls of AN women

**Figure 1:**
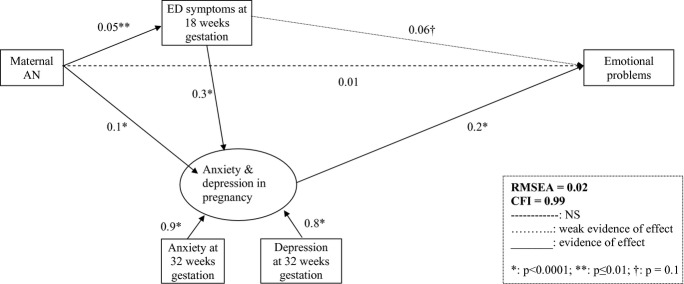
SEM model of emotional problems at 3½ years in girls of AN mothers.

The model that best fits the data (Figure 1) suggested an important role for pregnancy anxiety and depression mediating the effect of maternal AN and active ED pregnancy symptoms on emotional problems in girls. There was no evidence for a direct effect of maternal lifetime AN; the main effect of maternal lifetime AN was indirect via pregnancy ED symptoms and anxiety and depression. Child factors did not figure in the best-fitting model.

#### Conduct problems in girls of AN women

The best-fitting model was similar to the above model for emotional problems, although ED pregnancy symptoms did not directly affect conduct problems (direct path not statistically significant in this case) (see Figure S1).

#### Hyperactivity problems in girls of AN women

The best-fitting model was similar to the model for conduct problems (Figure S2).

#### Hyperactivity problems in girls of BN women

The model that best fits the data in this subset of analyses was similar to that obtained for hyperactivity in girls of women with AN: the effect of maternal lifetime BN on hyperactivity was indirect, via pregnancy anxiety and depression and ED symptoms (Figure S3).

### Boys

#### Emotional problems in boys of AN women

The model that best fits the data was similar to the model for emotional problems in girls of AN mothers: pregnancy maternal anxiety and depression mediated the effect of maternal lifetime AN and active ED pregnancy symptoms (see Figure S4). Child factors did not figure in the best-fitting model.

Given that the risk model in this instance was comparable to the risk model above in girls of women with AN (Figure 1); we were able to compare these two models. The comparison suggested no evidence that the best-fitting model was different for boys and girls (χ^2^ = 1.5, *df* = 3, *p* = .7).

#### Emotional problems in boys of BN women

**Figure 2:**
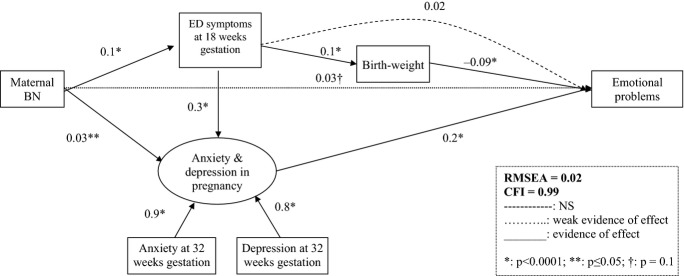
SEM model of emotional problems at 3½ in boys of BN mothers

In these analyses, the model that best fits the data suggested was weak evidence of a direct effect of maternal lifetime BN on emotional symptoms; this effect was mainly indirect, via pregnancy depression and anxiety. In this case, the effect of maternal lifetime BN via anxiety and depression was lower than that via active ED symptoms in pregnancy (total indirect effect via anxiety and depression = 0.01; total indirect effect via active ED symptoms in pregnancy = 0.02). Birthweight also mediated the effect of active ED symptoms on emotional problems (Figure 2). In particular, pregnancy ED symptoms in this group were positively associated with birthweight and birthweight was negatively associated with being more likely to have emotional symptoms. The best-fitting model did not include child factors (see Figure 2).

#### Conduct problems in boys of BN women

**Figure 3:**
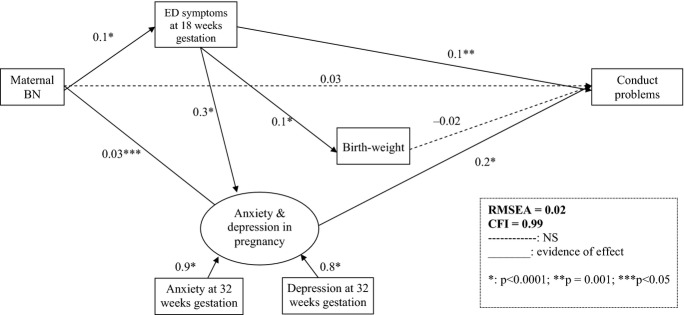
SEM model of conduct problems at 3½ in boys of BN mothers

As for emotional problems, we found that maternal lifetime BN mainly had an indirect effect on conduct problems; in particular, the strongest indirect effect was via active ED symptoms in pregnancy, although the effect was also mediated via pregnancy anxiety and depression. The best-fitting model for these analyses also did not include any child or postnatal factors (see Figure 3).

#### Multiple problems

As a post hoc set of analyses to determine whether the similarity in risk models for the psychopathology outcomes investigated (in particular, for girls of AN women and boys of BN women) was due to comorbidity, we investigated how many girls of women with AN and boys of women with BN who had multiple problems compared with unexposed using logistic regression.

Girls of women with AN were more likely to have two or more comorbid disorders compared with unexposed (OR = 2.6, 95% CI: 1.2–4.6, *p* = .02).

However, when girls with comorbid disorders were excluded from the SEM the fit of the models, the best-fitting model and the path coefficients did not change.

Boys of women with BN were more likely compared with the unexposed to have two or more comorbid problems (OR = 5, 95% CI: 2.3–10.8, *p* < .0001).

When boys with comorbid disorders were excluded from the SEM models the fit of the models, the best-fitting model and the path coefficients did not change.

## Discussion

### Risk for psychopathology in offspring of women with lifetime ED

Despite important limitations, previous research has suggested an increased risk for psychopathology (homotypic and heterotypic effects) in children of women with ED. We aimed to extend these findings using data from a large population-based study, and modeling the role of in utero and postnatal factors in increasing the risk for childhood psychopathology in children of ED women taking into account gender differences.

First, children of women with ED aged 3½ had increased odds of psychopathology across most domains (emotional, conduct, and hyperactivity) compared with unexposed children. Specifically, we highlighted a twofold increase in the odds for any psychopathology in children of mothers with ED. Moreover, we found evidence that girls of women with AN had a two and half-fold higher odds for having two or more comorbid problems. The same was true for boys of BN women who had a fivefold increased odds of having two comorbid mental health problems. Overall (across genders), an association between maternal ED (both AN and BN) and offspring emotional symptoms and maternal BN and conduct problems emerged from this study.

Although the literature in the field is scant, these results build on previous findings that maternal ED are associated with higher levels of psychopathology in the offspring (Reba-Harrelson et al., 2010; Stein et al., 1994).

### The role of antenatal and postnatal factors

Contrary to our expectation that the relationship between maternal ED and childhood psychopathology would include the interplay of pre and postnatal parental and child factors, this was not the case. Our findings were consistent with previous general findings on the major role of maternal anxiety and depression in the third trimester of pregnancy on childhood psychopathology (O’Connor et al., 2003; Rodriguez & Bohlin, 2005; Van den Bergh & Marcoen, 2004). We have previously shown that maternal antenatal depression and antenatal ED act in an additive way to predict perinatal anxiety and depression in this sample (Micali, Simonoff, & Treasure, 2011). The effect of maternal lifetime ED and active pregnancy ED symptoms on early childhood psychopathology was partly mediated by anxiety and depression at 32 weeks in pregnancy. This indirect effect was stronger for children of women with AN.

As expected, active ED symptoms were important mediators in relation to psychopathology; this was particularly true for children of women with lifetime BN.

Unfortunately, this study design did not allow testing whether the effect seen is purely due to fetal programming or genetic influences.

Postpartum anxiety and depression and parenting did not appear to be relevant for good fit of the models; nor did child factors (such as temperament and developmental level). This might be due to child factors being less potent, particularly at this age and to a strong in utero effect.

Importantly, our findings suggest some different risk pathways across gender and maternal diagnosis; for example, boys of women with BN appeared to be more susceptible to the effect of active pregnancy ED symptoms, both directly and via birthweight. In general, boys have been found to be more susceptible to in utero insults (Rutter et al., 2003). In contrast to this, both boys and girls of women with AN were more likely to have emotional symptoms and risk pathways did not differ. The mechanisms underpinning this finding and gender differences are exploratory and need further investigation.

Strengths of this study include the availability of a large sample of exposed women; moreover, the prospective nature of the study allows detailed investigation of causal inferences and clarification of timing of exposure.

Findings of this study need to be interpreted in light of relevant limitations. Information on childhood psychopathology was obtained from parents; this can introduce bias and misclassification of outcome. If this were the case, it is likely that the misclassification of outcome is differential, i.e., women with lifetime ED might be more likely to report problems in their children, and this might result in biased higher odds of psychopathology in the exposure groups compared with unexposed. Maternal history of ED was obtained by self-report; however, evidence suggests that self-report of ED is sensitive and specific (Keski-Rahkonen et al., 2007; Micali et al., 2012).

Although the SDQ has been recently used and validated in children <5 (Ezpeleta et al., 2013; Theunissen, Vogels, de Wolff, & Reijneveld, 2013), normative data are still limited in this age range. Lastly, although ALSPAC was shown to be largely representative of the local area, attrition was seen in this study with retention of older, more educated mothers; therefore, generalizability of our findings to other populations needs evaluating.

Due to the study design, it is difficult to disentangle how much of the effect seen is due to genetic or environmental factors or epigenetic effects, and future studies should focus on understanding these mechanisms.

Despite the limitations, our findings have important clinical implications. First, clinicians treating women with ED should be aware of the risk posed to the offspring. Anxiety and depression and ED in pregnancy are treatable and therefore negative child outcomes might be preventable.

These findings are indicative of shared liability to internalizing behaviors in children at risk for AN and externalizing behaviors in children at risk for BN, with a stronger association between maternal ED and emotional disorders in boys (compared with girls) and evidence of an association between maternal ED and hyperactivity problems in girls.

Investigating the longitudinal course of psychopathology in offspring of women with ED will shed more light on the long-term effects of maternal ED and on the underlying link between maternal ED and other psychopathology.

## Key points

- There is scant evidence that children of women with ED might be at risk for emotional problems.
- Emotional and behavioral problems in the context of maternal ED have not previously been studied in a large sample.
- This study highlights important differences in risk mechanisms for emotional and behavioral problems across genders.
- Pregnancy factors (ED symptoms, anxiety, and depression) predict psychopathology in children of women with ED.
- Differential risk related to specific ED has important implications for shared liability to psychiatric disorders.

## Supporting Information

**Figure S1** SEM model of conduct problems at 3½ in girls of AN mothers **Figure S2** SEM model of hyperactivity problems at 3½ in girls of AN mothers **Figure S3** SEM model of hyperactivity problems at 3½ in girls of BN mothers **Figure S4** SEM model of emotional problems at 3½ in boys of AN mothers
