# Supplementary material for: Childhood psychopathology in children of women with eating disorders: understanding risk mechanisms
Source: J Child Psychol Psychiatry. 2013 Jun 27;55(2):124–34. doi: 10.1111/jcpp.12112 (PMC4217387; doi:10.1111/jcpp.12112)
Supplement: Supplementary file 1 — Figure S1 SEM model of conduct problems at 3½ in girls of AN mothers Figure S2 SEM model of hyperactivity problems at 3½ in girls of AN mothers Figure S3 SEM model of hyperactivity problems at 3½ in girls of BN mothers Figure S4 SEM model of emotional problems at 3½ in boys of AN mothers [file jcpp0055-0124-sd1.docx]

**Supporting Information**

**Figure S1** SEM model of conduct problems at 3 ½ in girls of AN mothers
**Figure S2** SEM model of hyperactivity problems at 3 ½ in girls of AN mothers
**Figure S3** SEM model of hyperactivity problems at 3 ½ in girls of BN mothers
**Figure S4** SEM model of emotional problems at 3 ½ in boys of AN mothers
